# THE ASSOCIATION BETWEEN PHYSICAL FRAILTY AND DEPRESSIVE SYMPTOMS: DOES SOCIAL SUPPORT MATTER?

**DOI:** 10.1093/geroni/igz038.3546

**Published:** 2019-11-08

**Authors:** Shuting Liang, Dexia Kong, XinQi Dong

**Affiliations:** 1 Institute for Health, Health Care Policy & Aging Research, New Brunswick, New Jersey, United States; 2 Rutgers University, New Brunswick, New Jersey, United States

## Abstract

This research will present the association between physical frailty and depressive symptoms among U.S. Chinese older adults, and the extent to which social support moderates the relationship. Cross-sectional data were obtained from the Population Study of Chinese Elderly in Chicago collected between 2011 and 2013 (N=3,157). Physical frailty was assessed by the Short Performance Physical Battery (range=0-15). A cut-off point of 6 was used to define physical frailty as suggested by prior research. Depressive symptoms were assessed by the nine-item Patient Health Questionnaire. Social support was measured by a scale assessing positive support and negative strain from spouse, family members, and friends. Logistic regression analyses with interaction terms were conducted. In our sample, 1,682 (54.3%) had depressive symptoms, and 16.1% had physical frailty. Having physical frailty was positively associated with depressive symptoms (Odds Ratio [OR] 1.15, 1.11-1.18). Additionally, female gender (OR 1.39, 1.20-1.61), education (OR 1.03, 1.01-1.04), and chronic conditions (OR 1.18, 1.12-1.25) were positively associated with depressive symptoms. Social support (OR 0.85, 0.83-0.87) and children (OR=0.92, 0.87-.97) were negatively associated with depressive symptoms. Furthermore, family members (OR 0.96, 0.94-0.98) and friends (OR 0.96, 0.94-0.98) has moderating effect on the relationship between physical frailty and depressive symptoms. However, the interaction between social support from spouse and physical frailty was not significant. The findings highlight the interconnections among physical frailty, social support, and depressive symptoms. Intervention strategies focusing on social support may have the potential to reduce depressive symptoms among frail U.S. Chinese older adults.

